# MichelaNglo: sculpting protein views on web pages without coding

**DOI:** 10.1093/bioinformatics/btaa104

**Published:** 2020-02-15

**Authors:** Matteo P Ferla, Alistair T Pagnamenta, David Damerell, Jenny C Taylor, Brian D Marsden

**Affiliations:** b1 NIHR Oxford BRC Genomic Medicine, Wellcome Centre for Human Genetics, University of Oxford, Oxford OX3 7BN, UK; b2 Structural Genomics Consortium, University of Oxford, Old Road Campus Research Building, Oxford OX3 7DQ, UK; b3 Kennedy Institute of Rheumatology, University of Oxford, Oxford OX3 7FY, UK

## Abstract

**Motivation:**

The sharing of macromolecular structural information online by scientists is predominantly performed via 2D static images, since the embedding of interactive 3D structures in webpages is non-trivial. Whilst the technologies to do so exist, they are often only implementable with significant web coding experience.

**Results:**

Michelaɴɢʟo is an accessible and open-source web-based application that supports the generation, customization and sharing of interactive 3D macromolecular visualizations for digital media without requiring programming skills. A PyMOL file, PDB file, PDB identifier code or protein/gene name can be provided to form the basis of visualizations using the NGL JavaScript library. Hyperlinks that control the view can be added to text within the page. Protein-coding variants can be highlighted to support interpretation of their potential functional consequences. The resulting visualizations and text can be customized and shared, as well as embedded within existing websites by following instructions and using a self-contained download. Michelaɴɢʟo allows researchers to move away from static images and instead engage, describe and explain their protein to a wider audience in a more interactive fashion.

**Availability and implementation:**

Michelaɴɢʟo is hosted at michelanglo.sgc.ox.ac.uk. The Python code is freely available at https://github.com/thesgc/MichelaNGLo, along with documentations about its implementation.

## 1 Introduction

Communicating the interpretations of macromolecular experimental structures of proteins and their ligands to non-structural biologists is an important but non-trivial task. Providing static 2D images, no matter how visually appealing, inhibits the understanding of the 3D nature of the structural data. Yet, this approach is the current state-of-the-art within contemporary websites and digital publications because, whilst it is relatively trivial to embed two-dimensional images from visualization platforms into modern websites, it is entirely non-trivial to export and embed interactive 3D visualizations without significant experience of web technologies such as JavaScript, HTML and CSS.

A range of software applications that support the visualization of protein structures in three dimensions currently exist, either as commercial pieces of software or as freely available desktop applications (e.g. PyMOL, ICM-Browser etc.; Martinez *et al.*, 2019; Raush *et al.*, 2009; Schrödinger LLC, 2015) These standalone platforms are able to create interactive visualizations of structures, but the sharing of these as interactive views is often fundamentally locked into the ecosystem of the creator platform in terms of file formats, related costs and restrictive licencing terms. Previously, we developed the iSee platform (Lee *et al.*, 2009, 2011; Raush *et al.*, 2009) which for the first time made it possible to generate web-based interactive views of macromolecular structures whilst embedding them within text, making it possible for those with limited or no structural biology experience to better understand the information presented in such views. However, this platform depended upon a type of web-browser plugin which is no longer supported by mainstream browsers. More recently, a number of freely available JavaScript web-based protein structure 3D visualization libraries that can be embedded into websites have been created including NGL (Rose and Hildebrand, 2015), Mol* (Sehnal *et al.*, 2018), PV (Guex *et al.*, 2009) and JMol (Hanson and Lu, 2017). However, there is still a significant barrier associated with the creation of the interactive visualizations for embedding in websites either from the ground-up or via the use of widely adopted desktop applications such as PyMOL.

We present Michelaɴɢʟo (https://michelanglo.sgc.ox.ac.uk), a web application which attempts to lower the barrier to generation and delivery of basic interactive 3D macromolecular visualizations for web pages without the need to write any JavaScript.

## 2 Implementation

Michelaɴɢʟo supports the creation of shareable, customizable and secure web pages that use the NGL library (Rose and Hildebrand, 2015) to display the protein interactively alongside text annotated with hyperlinks that control the visualization so that it may reflect the context of the text it is linked to, thereby providing the ability to provide an interactive narrative alongside a responsive 3D view. As sources of initial structural data, Michelaɴɢʟo is able to convert PyMOL PSE files to NGL implementations, preserving the majority of graphical features. Alternatively, PDB files or PDB codes can be used as starting points. Furthermore, gene symbols or protein names can be entered, providing a summary of structural coverage for the associated proteins (featureViewer code from Zahn-Zabal *et al.* 2020) thereby allowing the user to make informed decisions on which PDB structures to pick to form the basis of the required visualization.

Basic tools to modify the representation and orientation of the protein and its ligands are available within the interface, but it is important to note that Michelaɴɢʟo is not intended to be a replacement for fully featured applications such as PyMOL. Once a visualization is generated, instructions are provided on how to implement the interactive view in a web page (e.g. departmental webpage or blogposts) and a complete HTML file can also be downloaded for use as supplementary material, e.g. The title, description (in markdown format), layout, security and editing rights of the resulting page can be customized.

**Fig. 1.**
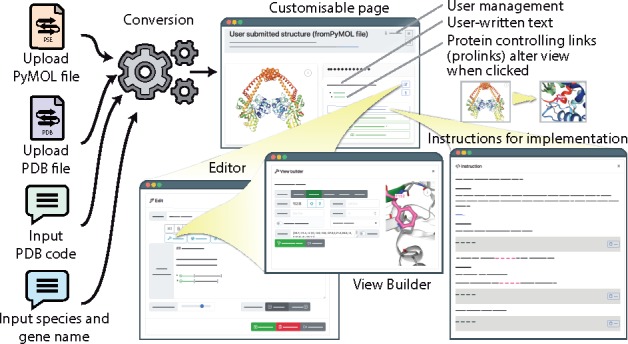
From model to webpage. There are four input routes (PyMOL file, PDB file, PDB code and protein name), which generate a page that can be further customized by editing the description and by adding protein controlling links within the description via the view builder. Once a satisfactory page has been made, the user can share the URL of the page, download a static copy or follow the detailed instructions on implementing the view on their own site

An important feature within Michelaɴɢʟo is the ability to create ‘prolinks’, protein-view-controlling links that are implemented as hyperlinks within user-provided text. These links are specified via HTML syntax and can be created via the user interface (Fig. 1). Examples of the capabilities of prolinks include changing the camera orientation, highlighting residues or regions, changing which chains are visible and additionally the detection and visualization of clashes as spinning spiky balls, similarly to Foldit (Kleffner *et al.*, 2017) or ICM-Browser.

To support the interpretation of non-synonymous genetic variants on protein structure and function, Michelaɴɢʟo is able to take a list of point mutations, generating different models showing their location in the structure and combining these visualizations into a single web page.

Static 2D images of proteins in publications and websites are often annotated and sometimes contain inset images or plots. Michelaɴɢʟo can be used in combination with such a ‘front’ image whereby the web page loads with the given image but when clicked the image is replaced with the interactive 3D view. This allows Michelaɴɢʟo to be easily incorporated into existing web pages or digital publications with no reduction in fidelity, whilst still providing the option for 3D views to be activated. Additionally, different models and their representations can be combined into a single page wherein prolinks optionally control which model to show.

The use of Michelaɴɢʟo as a tool to share interactive visualizations does not depend upon access to a website under one’s own control. Michelaɴɢʟo is able to host completed visualizations which can then be shared privately by using the provided URL which contains a unique identifier and is well-suited even for unpublished structures. To support this, users must register for an account allowing them to edit the description, keep track of the pages they create and to control who can edit them. The permissions can also be altered, ranging from making the page publicly listed to encrypting the model and description without storing the password anywhere on the server.

A significant challenge associated with the publishing of digital content is its sustainability and fidelity over time. To combat this problem, a monitoring system for pages flagged by the user as requiring protection is in place wherein every month, and at every codebase update, the visualizations generated from each link in the protected page are compared with a reference image in order to guarantee that the generated views will remain unchanged, therefore allowing pages in Michelaɴɢʟo to be safely cited in publications.

The code for Michelaɴɢʟo is publicly hosted at github.com/thesgc/MichelaNGLo, under an MIT licence, allowing others to use and improve the platform. Tutorials on usage can be found in the pages and video descriptions are available in the documentation.

## 3 Conclusions

The aim of Michelaɴɢʟo is to facilitate the creation of interactive web pages by geneticists and structural biologists. Several examples can be found at michelanglo.sgc.ac.uk/gallery. Within NIHR Oxford BRC, the use of Michelaɴɢʟo has proven far more effective than text and static images to describe the effects of a range of mutations found in a next generation sequencing cohort, WGS500 (Taylor *et al.*, 2015). A case example is LZTR1 (michelanglo.sgc.ox.ac.uk/data/LZTR1). In this protein, the mutations result in Noonan syndrome, but the location of these result in different modes of inheritance: mutations on one face of the protein—presumably where its target (hRAS) binds—are dominantly inherited, whilst mutations elsewhere are recessive and destabilizing (Motta *et al.*, 2019; Pagnamenta *et al.*, 2019). The ability to interact with the protein’s structure in three dimensions better imparts this observation, especially being able to focus on recessive mutations to see the clashes (likely destabilization) or dominant mutations to see whether they cluster on the same face or close to a known phosphorylation site, for example.

Michelaɴɢʟo, by lowering the barrier to implementation and adoption of 3D interactive visualizations, is a useful basic tool for bridging the gap between web developers and researchers. We hope that it will encourage the move away from static images as the default communications modality and towards interactive protein visuals by default in digital publications.
